# Phytic acid attenuates acetaminophen-induced hepatotoxicity via modulating iron-mediated oxidative stress and *SIRT-1* expression in mice

**DOI:** 10.3389/fphar.2024.1384834

**Published:** 2024-05-01

**Authors:** Hend M. Hassan, Nehal H. M. Abdel-Halim, Ibrahim El-Shenbaby, Manar A. Helmy, Maha O. Hammad, Ola A. Habotta, Eman M. El Nashar, Mansour A. Alghamdi, Rashid A. Aldahhan, Khulood M. Al-Khater, Basmah Almohaywi, Eman A. E. Farrag

**Affiliations:** ^1^ Human Anatomy and Embryology Department, Faculty of Medicine, Mansoura University, Mansoura, Egypt; ^2^ Human Anatomy and Embryology Department, New Mansoura University, New Mansoura, Egypt; ^3^ Physiology Department, Faculty of Medicine, Mansoura University, Mansoura, Egypt; ^4^ Clinical Pharmacology Department, Faculty of Medicine, Mansoura University, Mansoura, Egypt; ^5^ Forensic Medicine and Toxicology Department, Faculty of Medicine, Mansoura University, Mansoura, Egypt; ^6^ Medical Biochemistry and Molecular Biology Department, Faculty of Medicine, Mansoura University, Mansoura, Egypt; ^7^ Forensic Medicine and Toxicology Department, Faculty of Veterinary Medicine, Mansoura University, Mansoura, Egypt; ^8^ Department of Anatomy, College of Medicine, King Khalid University, Abha, Saudi Arabia; ^9^ Genomics and Personalized Medicine Unit, The Center for Medical and Health Research, King Khalid University, Abha, Saudi Arabia; ^10^ Department of Anatomy, College of Medicine, Imam Abdulrahman Bin Faisal University, Dammam, Saudi Arabia; ^11^ Department of Pharmaceutical Chemistry, College of Pharmacy, King Khalid University, Abha, Saudi Arabia

**Keywords:** acetaminophen, phytic acid, iron, *SIRT*-1, *CYP2E1*, liver

## Abstract

**Introduction:** Administration of high doses of acetaminophen (APAP) results in liver injury. Oxidative stress and iron overload play roles in the pathogenesis of APAP-induced hepatotoxicity. The present study assessed the potential hepatoprotective effects of phytic acid (PA), a natural antioxidant and iron chelator, on APAP-induced hepatotoxicity and the possible underlying mechanism through its effects on *CYP2E1* gene expression, iron homeostasis, oxidative stress, and *SIRT*-1 expression levels.

**Methods:** Twenty-four adult male albino mice were used in this study. Mice were divided into four groups (six mice in each group): control, APAP-treated, PA-treated and APAP + PA-treated groups. Liver function tests, serum and liver tissue iron load were evaluated in all the study groups. Hepatic tissue homogenates were used to detect oxidative stress markers, including malondialdehyde (MDA) and reduced glutathione (GSH). Histological hepatic evaluation and immunohistochemistry of *SIRT*-1 were performed. Quantitative real-time PCR was used for the assessment of *CYP2E1* and SIRT-1 gene expressions. APAP-induced biochemical and structural hepatic changes were reported.

**Results:** PA administration showed beneficial effects on APAP-induced hepatotoxicity through improvements in liver functions, decreased *CYP2E1* gene expression, decreased serum and liver iron load, decreased MDA, increased GSH, increased *SIRT*-1 expression level and improvement in hepatic architecture.

**Conclusion:** Conclusively, PA can be considered a potential compound that can attenuate acetaminophen-induced hepatotoxicity through its role as an iron chelator and antioxidant, as well as the up-regulation of *SIRT*-1 and down-regulation of *CYP2E1.*

## Introduction

The liver is a major site of metabolism and detoxification of drugs and xenobiotics in the human body (Cichoż-Lach and Michalak, 2014). Over half of all cases of liver disorders are induced by excessive or improper drug use (Li et al., 2016). Acetaminophen (APAP) is a common analgesic and antipyretic drug that is regarded as a safe medication at therapeutic doses (Li et al., 2016). Even though it is a widely used over-the-counter drug, it accounts for most cases of drug-induced liver injury (Fontana, 2008). On administering high doses of APAP (a single dose of 6-7 g or 4 g/day for two successive days in adults), the sulfation and glucuronidation pathways become saturated. On contrary, oxidation by cytochrome P450 family 2 subfamily E member 1 (*CYP2E1*) increases, with an increase in the formation of a highly reactive toxic intermediate metabolite, N-acetyl-p-benzoquinone imine (NAPQI) (Fontana, 2008). In therapeutic doses, NAPQI is detoxified by reduced glutathione (GSH) (Wang et al., 2017). Excessive NAPQI production in toxicity depletes GSH stores in the liver. NAPQI covalently binds to sulfhydryl groups in cellular proteins, especially mitochondrial proteins. This results in mitochondrial oxidative stress and dysfunction, ultimately leading to hepatocyte necrosis (Yan et al., 2018).

According to (Zhang et al., 2012), oxidative stress, protein oxidation, membrane lipid peroxidation, and liver tissue damage are thought to be the main mediators of APAP-induced liver injury. However, the exact intracellular mechanism that contributes to inducing this oxidative stress is not completely understood (van Swelm et al., 2012).

Iron has been discovered to play a part in APAP-caused hepatotoxicity. Ferrous iron is released into the cytosol from ruptured lysosomes following exposure to a hazardous dose of APAP, which induces the production of reactive oxygen species in what is called the Fenton reaction. This starts the opening of the mitochondrial permeability transition pore, which sets off a chain of events that destroy liver cells (Kon et al., 2010; Jomova and Valko, 2011). Because it is the main organ for storing iron, the liver is extremely susceptible to tissue damage from iron (Zhang et al., 2012).

The sirtuin family member *SIRT-1* is a protein lysine deacetylase that regulates several physiological processes, including energy production, inflammatory responses, cell survival, DNA repair, tissue regeneration, and stress responses. *SIRT-1* inducers are hepatoprotective agents (Farghali et al., 2019). Activation of *SIRT*-1 can protect the cells against death by several mechanisms, and one of the suggested mechanisms is its ability to inhibit ferroptosis; iron-mediated programmed cell death that is characterized by oxidative cell death resulting from iron-dependent accumulation of lipid reactive species. Activating *SIRT*-1 can reduce iron accumulation by inhibiting hepcidin. This mechanism suggests that SIRT1 may inhibit ferroptosis by reducing intracellular iron levels. Moreover, *SIRT*-1 reduces ferroptosis by activating autophagy (Su et al., 2021).

N-acetylcysteine is the cornerstone treatment for hepatotoxicity following APAP overdose; however, it has certain limitations in terms of side effects and a limited therapeutic window (Bateman et al., 2014). Natural compounds derived from herbal plants have gained scientific interest as a new promising path to treat liver injuries (Al-Qarawi et al., 2005).

As a continuation to researches concerned with natural compounds, phytic acid (PA) was evaluated in this study in APAP induced hepatoxicity based on its antioxidant properties and its ability to modulate iron metabolism. PA is a major phosphorus storage compound in most seeds and cereal grains (Diouf-Lewis et al., 2017). PA is generally referred to as an anti-nutrient due to the food micronutrients chelating ability, making them unabsorbable and thus low bioavailability (Thavarajah et al., 2010). However, PA when administered in drinking water is rapidly absorbed through the stomach and upper small intestine, becomes quickly dephosphorylated within the mucosal cells and is distributed to various organs distributed to various organs as early as l h following intragastric administration (Sakamoto et al., 1993). Studies indicate that PA has no cytotoxic effect but high intake of foods rich in PA should be considered to minimize impact on mineral absorption (Song et al., 2019).

The ability of PA to chelate iron and remove it from the circulation, together with its ability to prevent the production of iron-driven hydroxyl radicals and reduce lipid peroxidation, contribute to its antioxidant activity. Additionally, it has been proposed that PA may directly bind a few enzymes throughout the process, changing the way those enzymes act (Omoruyi et al., 2013). Phytic acid was also reported to induce *SIRT-1* activity in different tissues, which can control iron metabolism in tissues (Zhang and Tang, 2023). Increasing data suggests that PA has a positive impact on several conditions, indicating that it has a great potential for usage in a variety of applications. Several studies have been done, both *in vitro* and *in vivo*, showing strong anti-cancer activity evidence for PA. More different studies showed its beneficial effects on several other diseases such as diabetes, vascular calcifications and consequently reduced risk of cardiovascular events, coronary heart disease, Parkinson’s disease, and kidney stones treatment. However, more intensive studies are needed to gain a better understanding of the mechanisms of action (Bloot et al., 2023).

The present study aimed to assess the potential therapeutic effects of PA on APAP-induced hepatotoxicity and the possible underlying mechanisms through its effects on *CYP2E1* gene expression, serum and tissue iron load, oxidative stress and *SIRT-1* expression.

## Methods

### Animals

The study was carried out on twenty-four male albino mice weighing 26–28 g. Throughout the typical day-night cycle (12 h–12 h), they had unlimited access to food and drink. At the Faculty of Veterinary Medicine, the mice were housed under veterinary care, and their suffering was minimized as much as possible.

### Study design

**FIGURE 1:**
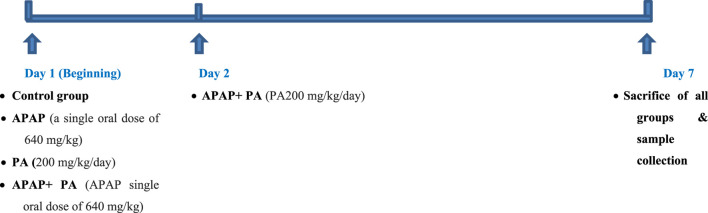
The study design. Mice were given a single oral dose of APAP and PA was given for 6* *days.

The studied mice were randomly allocated into 4 groups (6 mice in each group); control group: received distilled water orally via gastric gavage for 7 days; APAP-treated group: received APAP at a single oral dose of 640 mg/kg, dissolved in distilled water (Singh et al., 2011); PA-treated group: received PA at a dose of 200 mg/kg/day, dissolved in distilled water orally via gastric gavage for 7 days (Bhowmik et al., 2017); and APAP + PA treated group: received a single oral dose of APAP followed by PA for 6 days (Singh et al., 2011; Bhowmik et al., 2017) (Figure 1).

### Samples collection and storage

To confirm APAP toxicity, 0.3 mL of blood were collected from the tail vein 4 hours after administering the APAP dose. At the end of the experiment, it is sacrification was performed by decapitation. Blood samples were collected at the end of the experiment under anesthesia via cardiac puncture in EDTA-free tubes. The tubes were left to clot for 10 min, centrifuged for serum and plasma separation and stored at −20°C.

The liver was carefully dissected and cut into three pieces. The first piece was kept in 10% buffered formalin for paraffin block formation. Four µm thick sections were obtained and stained with hematoxylin and eosin (H & E) and Perl’s Prussian blue stains. Additionally, immunostaining was done on hepatic sections for the *SIRT*-1 marker. The second piece of hepatic tissues (about 50 mg) was collected and completely submerged in RNA later reagent (Qiagen, Germany), with approximately 500 µL of reagent per 50 mL of tissue. Then it was incubated at 2°C–8°C for 1 day, followed by storage at −80°C for subsequent mRNA quantification. The third piece was kept fresh for hepatic homogenate preparation.

### Estimation of liver function parameters

Aspartate aminotransferase (AST), alanine aminotransferase (ALT) were assayed in serum samples obtained from all groups of mice by a colorimetric method of Reitman and Frankel (Reitman and Frankel, 1957), while albumin was assessed according to the method of Pinnell and Northam (Pinnell and Northam, 1978) using commercially available kits (Diamond Diagnostic, Egypt) and the Erba CHEM-7 device (ERBA Diagnostics, India).

### Estimation of iron parameters

Serum iron (SI) concentration and total iron-binding capacity (TIBC) were estimated by colorimetric analysis according to the manufacturers’ protocols for commercial kits (Biodiagnostic, Egypt).

### Estimation of APAP levels in serum using high-performance liquid chromatography (HPLC)

To confirm obtaining toxic levels, an estimation of the APAP level in serum was performed. For extraction, in 15-mL screw-cap tubes with Teflon-lined caps, 0.5 mL of serum or working standards were put in. Each tube received 0.25–0.5 g of solid NaCl as well as 5 mL of diethyl ether. The tubes were shaken 50–100 times, then the phases were separated with a quick centrifugation. The ether phase was transferred to a conical centrifuge tube, where it was vaporized at room temperature while being blown by air. The sample was constructed into the mobile phase and 5 μL were injected into the system. Chromatographic analysis was performed using HPLC Agilent technology, 1260 series, using Poroshell 120 EC-C18. Sample: 4.6 × 50 mm, 2.7 μm The temperature was 40°C. The mobile phase consisted of water, 0.1% formic acid and methanol at a flow rate of 1 mL/min. Injection volume detection was performed using DAD at a wavelength of 254 nm. Blank serum samples were spiked with five different concentrations of the acetaminophen standard. These samples were extracted according to the described method and then injected into the HPLC. Chromatogram results were used for the formation of calibration curves for the measured analytes. The calibration curve was linear, with a correlation coefficient of 0.99223.

### Colorimetric assay of MDA and GSH in hepatic tissue homogenate

Oxidative stress was evaluated by assaying reduced glutathione (GSH) and malondialdehyde (MDA) content in hepatic tissue homogenates using colorimetric kits (Biodiagnostic, Egypt) and following the instructions of the manufacturer. The tissues were blotted on filter paper, weighed, and homogenized in 0.05 M phosphate buffer (pH7.5) using a tissue homogenizer at 40°C. The homogenate was centrifuged at 10,000 rpm for 20 min and clear supernatants were collected and stored on ice until the time of the assay. GSH level was assayed based on the method of Beutler et al. (Beutler et al., 1963). The reaction of GSH and 5,5′dithiobis (dithionitrobenzoic acid) (DTNB) generated a reduced yellow-coloured chromogen. The concentration of reduced chromogen is directly proportional to that of GSH. The absorbance was estimated spectrophotometrically at 405 nm. The level of MDA, an indicator of lipid peroxidation, was estimated by the method of Ohkawa et al. (Ohkawa et al., 1979). The principle depends on the reaction between MDA and thiobarbituric acid (TBA) in an acidic medium at a temperature of 95°C for 30 min to generate the pink TBA-reactive substances (TBARS). The absorbance of the pink products can be read spectrophotometrically at 535 nm.

### mRNA quantification by quantitative real-time PCR (qRT-PCR)

Total RNA was isolated using QIAzol-Reagent (Qiagen, Germany), based on modified Chomczyński & Sacchi’s method (Chomczynski and Sacchi, 2006), following the manufacturer’s protocol. The amount and purity of isolated RNA were estimated by a NanoDrop Spectrophotometer (Thermo Fisher Scientific, USA) via optical density measurements at 260 and 280 nm. One thousand ng of total RNA was reverse transcribed into complementary DNA (cDNA) using the SensiFAST cDNA Synthesis Kit (Bioline, UK), following the manufacturer`s instructions. The cDNA templates were applied to a real-time PCR instrument (Applied Biosystems 7500, United States) for amplification using specific primers for *SIRT-1* and *CYP2E1*. The sequences of the primer sets for *SIRT-1, CYP2E1* and glyceraldehyde-3-phosphate dehydrogenase (GAPDH) were designed using Primer3 software (v.4.1.0; http://primer3.ut.ee) and supplied as follows: for *SIRT-1* (MusMusculus; PCR amplicon: 137bp; RefSeq: NM_019812.3): F: 5′-GGC​CGC​GGA​TAG​GTC​CAT-3′ and R: 5′-AAC​AAT​CTG​CCA​CAG​CGT​CA-3′; for *CYP2E1* (MusMusculus; PCR amplicon: 207bp; RefSeq: NM_021282.3): F: 5′-ATG​TCA​TCC​CCA​AGG​GTA​CAG-3′ and R: 5′-GCA​GAA​ACA​GTT​CCA​TGC​GG-3′; and for GAPDH (MusMusculus; PCR amplicon: 123bp; RefSeq: NM_001289726.1): F: 5′-AGG​TCG​GTG​TGA​ACG​GAT​TTG-3′ and R: 5′-TGT​AGA​CCA​TGT​AGT​TGA​GGT​CA-3′. The expressions of *SIRT-1* and *CYP2E1* were normalized to the expression of the endogenous control GAPDH. The 20 μL reaction mixture was prepared as HERA SYBR green PCR Master Mix (2X) (Willowfort, UK), 2 μL of cDNA fragments, 1 μL (10 pmol) of each primer and 6 μL of nuclease-free water. The reaction mixture was applied to a real-time PCR apparatus (Applied Biosystems 7500, USA). The thermal program was as follows: initial denaturation at 98°C for 3 min and 40 cycles (95°C/10 s, 60°C/30 s). The cycle threshold (CT) for the target genes (*SIRT-1* and *CYP2E1*) and the endogenous control GAPDH for each sample were detected and the relative quantitation of *SIRT-1* and *CYP2E1* mRNA expression levels was estimated by normalizing CT of each gene with the CT of GAPDH in each sample using the 2^−ΔΔCT^ method (Livak and Schmittgen, 2001).

### Hematoxylin and eosin stain (H& E)

This stain was used for evaluation of liver architecture, inflammatory cell infiltration, vascular congestion and hepatocyte necrosis, apoptosis, steatosis, vacuolation, edema and malignant changes (Bancroft and Gamble, 2008). We evaluated the histopathological changes represented in vacuolations, congestion, inflammation and necrosis in all studied groups under light microscopy with photomicrographs (x 400). A semiquantitative score was given based on the absence (=0) or presence (=1) of each of the histopathological finding. The evaluation was represented as the sum of all observed changes.

### Perl’s prussian blue stain

This stain was used for detection of iron in hepatic tissue. The method stains mostly iron in the ferric state, which includes ferritin and hemosiderin, rather than iron in the ferrous state (Iezzoni, 2018).

### Immunohistochemical detection of *SIRT*-1 in hepatic tissue

In order to stop endogenous peroxidases, 0.03% H_2_O_2_ was utilized. The antigens were first prepared in buffered saline containing 5% bovine serum albumin for 20 min in a microwave with sodium citrate buffer (pH = 6). Sections were then treated for a whole night at 4°C with a primary antibody against *SIRT*-1 (1:500, ab189494). The avidin-biotinylated peroxidase complex (ABC-kit) and DAB substrate (ab64238) were used in accordance with the manufacturer’s instructions to detect the reaction. After slices were dehydrated in progressively stronger alcohols, cleaned in xylene, and mounted, hematoxylin was used as a counterstain (Chen et al., 2010).

### Measurement of the percentage area of a positive *SIRT*-1 immunoreaction in hepatic tissue

Images were taken using a digital camera (Toucan type BX53, Japan) linked to a computer and a light microscope (Olympus model BX53, Tokyo, Japan). For each mouse in each group, the liver was examined in five evenly spaced, 4 µ thick sections using a 40x lens and a ×400 magnification (area: 0.071 mm2). The area fraction of *SIRT-1* immune expression was the calculated measure. Using ImageJ (Fiji, version 1.48), a computerized image analysis was taken. The pigmentation that was indicative of immune expression was brownish. Three different colored images were created using a color deconvolution plug-in and the H-DAB-vector as the selected color: green, brown, and blue. The DAB images (brown in color) were calibrated using area fraction (Hassan et al., 2022). The threshold was adjusted for more precision.

### Statistical analysis

The data were statistically analyzed using IBM SPSS, version 26.0. The results of the Shapiro test for normality demonstrated that the data were distributed normally. Data were expressed as mean ± SD and a 0.05 level was used to determine the significance. The one-way ANOVA test was used to compare the means of the 4 study groups and the post-hoc games-Howell test was performed to determine the pairwise comparisons.

## Results

### Effect of PA on liver function parameters (ALT, AST and albumin)

Compared to control group, mice treated with APAP exhibited increased levels of liver enzymes and decreased albumin levels (*p* ≤ 0.001). The PA + APAP group produced a statistically significant decrease in the liver enzymes and an increase in the albumin level when compared to the APAP group (*p* ≤ 0.001) (Table 1).

**TABLE 1:** Effect of phytic acid on liver function assessment in mice serum of different study groups.

Groups	ALT (U/L)	*p*-Value	AST (U/L)	*p*-Value	Albumin (g/dL)	*p*-Value
Control (*n* = 6)	106.00 ± 9.86		235.00 ± 24.01		3.11 ± 0.18	
APAP (*n* = 6)	187.83 ± 28.5^a^	≤0.002*	438.00 ± 64.89^a^	≤0.001*	2.43 ± 0.17^a^	≤0.001*
PA (*n* = 6)	100.33 ± 10.5	= 0.7	215.50 ± 22.46	= 0.5	3.29 ± 0.08	= 0.2
APAP + PA (*n* = 6)	124.67 ± 3.2^b^	≤0.01*	331.00 ± 37.13^b^	≤0.001*	2.75 ± 0.15^b^	≤0.001*

Data were presented as mean ± SD (6 mice in each group). Comparison between groups was performed via One way ANOVA followed by *post hoc* Games Howell test, significant *p*-values (≤0.05). * Represented significance. a: Significance between the control and APAP, groups. b: Significance between the APAP and APAP + PA groups.

Abbreviations: APAP, acetaminophen; PA, phytic acid; ALT, alanine transaminase; AST, aspartate transaminase.

### Effect of PA on serum iron level and TIBC

Compared to the control group, mice treated with APAP exhibited an increased level of serum iron and a decreased TIBC level (*p* ≤ 0.001). The PA + APAP group produced a statistically significant decrease in serum iron and increase in TIBC when compared to the APAP group (*p* = 0.02 and 0.01, respectively) (Table 2).

**TABLE 2:** Effect of phytic acid on serum iron and TIBC among different experimental groups.

Groups	TIBC (μmol/L)	*p*-Value	Iron (μmol/L)	*p*-Value
Control (*n* = 6)	479.00 ± 18.62		496.50 ± 26.84	
APAP (*n* = 6)	353.67 ± 29.26^a^	≤0.001*	685.00 ± 45.18^a^	≤0.001^*^
PA (*n* = 6)	499.00 ± 15.34	= 0.3	474.00 ± 17.53	= 0.2
APAP + PA(*n* = 6)	394.67 ± 21.99^b^	≤0.01*	608.67 ± 49.99^b^	≤0.02*

Data are presented by mean ± SD (6 mice in each group). Comparison between groups was performed via One way ANOVA followed by *post hoc* Games Howell test, significant *p* values (≤0.05). * Represented significance. a: Significance between the control and APAP groups. b: Significance between the APAP and APAP + PA groups. Abbreviations: APAP; acetaminophen, PA, phytic acid; TIBC, total iron binding capacity.

### Serum level of the APAP using HPLC

The estimated APAP level in the APAP group was 29.387 ± 2.3 μg/mL, 4 hours after exposure to toxic levels in the APAP group and 27.567 ± 1.9 μg/mL in the APAP + PA group before administrating PA. There was no statistically significant difference between these two values.

### Effect of PA on oxidative stress markers

The APAP treatment induced a statistically significant increase in MDA level and a decrease in GSH level in mice liver (*p* ≤ 0.001), while PA produced an improvement in the oxidative stress state by decreasing the MDA level and increasing the GSH level in the APAP + PA group when compared to the APAP group (*p* ≤ 0.001) (Table 3).

**TABLE 3:** Effect of phytic acid on oxidative stress markers in mice liver homogenate.

Groups	MDA (nmol/g)	*p*-Value	GSH (mmol/mg)	*p*-Value
Control (n = 6)	4.98 ± 0.40		1.08 ± 0.10	
APAP (n = 6)	11.03 ± 1.06^a^	≤0.001*	0.59 ± 0.13^a^	≤0.001*
PA (n = 6)	3.79 ± 0.40	≤0.001*	1.29 ± 0.06	≤0.001*
APAP + PA(n = 6)	5.96 ± 1.00^b^	≤0.001*	0.94 ± 0.09^b^	≤0.001*

Data are presented by mean ± SD (6 mice in each group). Comparison between groups was performed via One way ANOVA followed by *post hoc* Games Howell test, significant *p* values (≤0.05). * Represented significance. a: Significance between the control and APAP, groups. b: Significance between the APAP and APAP + PA groups. Abbreviations: APAP, acetaminophen; PA, phytic acid; MDA, malondialdehyde; GSH, reduced glutathione.

### Effect of PA on *SIRT*-1 and *CYP2E1* mRNA levels

For the effect of PA on the regulation of *SIRT-1* transcriptional level, there was a statistically significant decrease in *SIRT-1* mRNA level in mice liver in the group treated with APAP when compared to the control group (*p* ≤ 0.001). In addition, *SIRT-1* mRNA level increased significantly in the APAP + PA group when compared to the control group (*p* ≤ 0.001).

**FIGURE 2:**
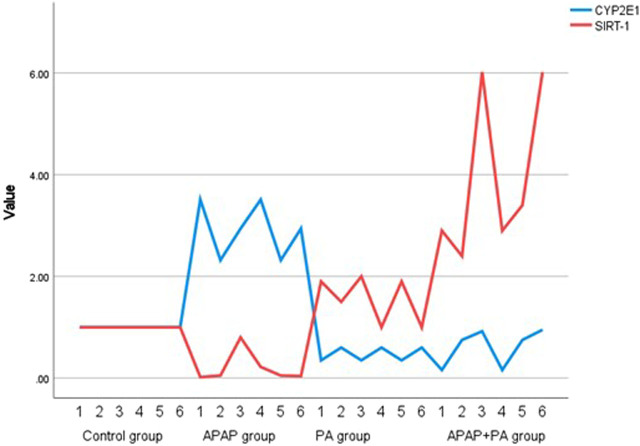
Multiple line graph for individual values of *CYP2E1* and *SIRT*-1 in each group (6 mice in each group). Comparison between groups was performed via one way ANOVA followed by a post-hoc Games-Howell test; significant p-values (≤ 0.05). Abbreviations: SIRT-1, sirtuin-1; CYP2E1, cytochrome P450 family 2 subfamily E member 1.

Regarding *CYP2E1* mRNA level, it increased in the APAP group when compared to the control group (*p* ≤ 0.001). Phytic acid alone decreased *CYP2E1* mRNA level with a statistically significant difference compared to the APAP group (*p* ≤ 0.001), but without a statistically significant difference when compared to the control group. The APAP + PA group showed a statistically significant decrease when compared to the APAP group (*p*-value = 0.01), but it does not reach the control level (Figure 2).

We used the comparative delta-Ct method to ensure that GAPDH expression is consistent throughout the samples and groups. In the control group, the Ct mean of GAPDH was 20.8 (SD = 1.51), while the Ct mean of GAPDH in PA group was 20.43 (SD = 1.13). The Ct mean of GAPDH in the APAP group was 19.4 (SD = 1.47), while Ct mean in the APAP + PA group was 20.2 (SD = 1.14).

### Results of hematoxylin and eosin staining of hepatic specimens

**FIGURE 3:**
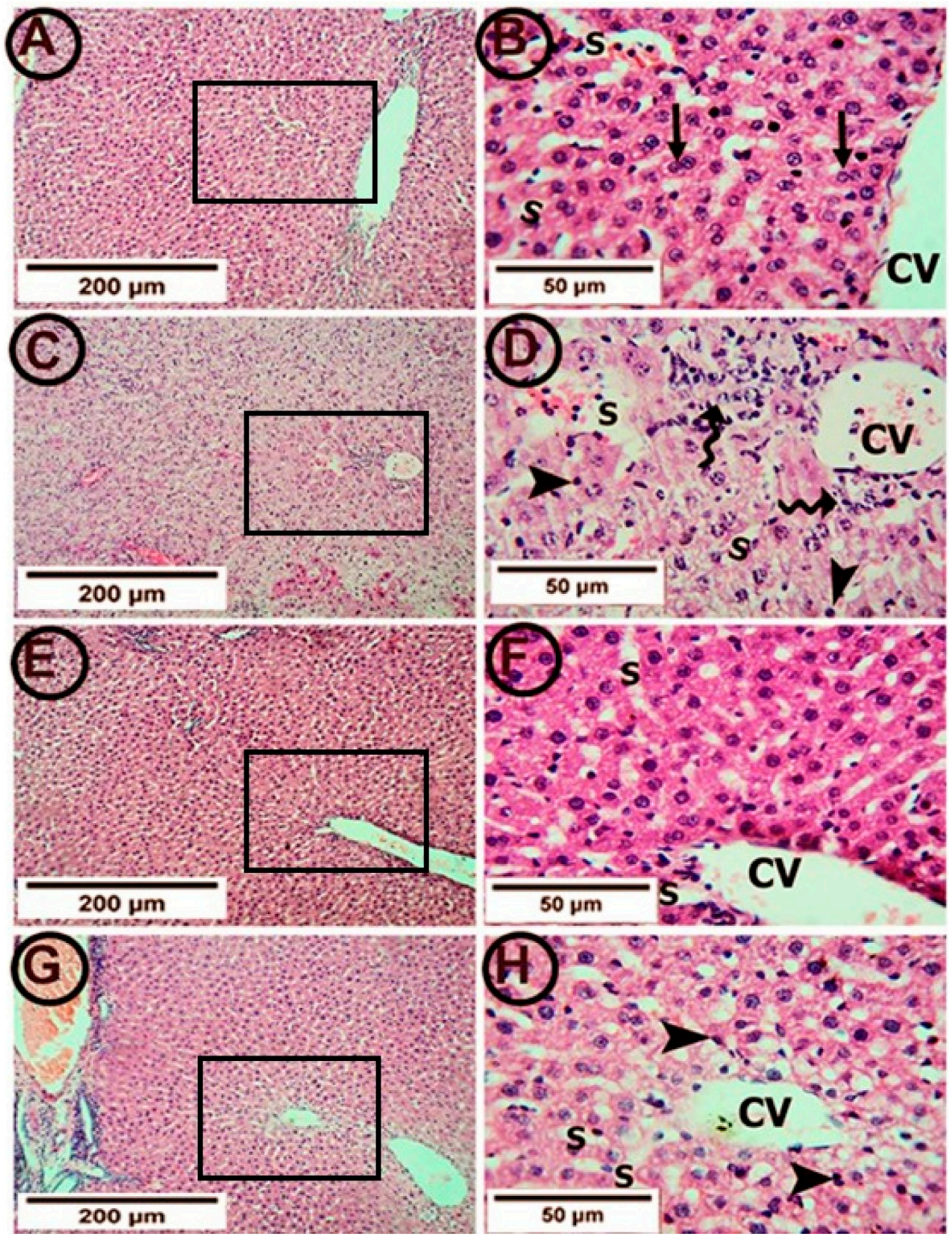
Photomicrographs of hepatic tissue in the study groups (6 mice in each group). **(A,B)** the control group showed a normal liver architecture with hepatocytes radiating from the central vein (CV). The individual cell appeared polyhedral with acidophilic granular cytoplasm. Binucleated cells were common (arrows). Hepatic sinusoids (S) appeared as narrow spaces between the hepatic plates. **(C,D)** The APAP group showed a congested central vein (CV) with perivascular lymphocytic infiltration (tailed arrows). Most hepatocytes appeared with deeply stained nuclei (arrowheads) and vacuolated cytoplasm. The sinusoids (S) were congested. **(E,F)** the PA group showed clear central veins (CV) and sinusoids (S). The hepatocytes appeared normal at the cytoplasmic and nuclear levels. **(G,H)** the APAP + PA group showed minimal congestion of the central veins (CV). Many of the hepatocytes had the cytoplasm and nuclei with normal appearance. However, the perivascular cells still showed vacuolated cytoplasm with darkly stained nuclei (arrow heads). No lymphocytic infiltration was seen. (H & E: A, C, E, G X 100–B, D, F, H X 400). Abbreviations: APAP; acetaminophen, PA, phytic acid.

The control group showed a normal hepatic architecture (Figures 3A, B). The hepatocytes were arranged in the form of plates radiating from the central vein. Each hepatocyte appeared polyhedral with acidophilic granular cytoplasm. Binucleated cells were common. Hepatic sinusoids appeared as narrow spaces between the hepatic plates. On the other hand, treatment with APAP showed pathological changes in the form of a congested central vein with perivascular lymphocytic infiltration. Most hepatocytes appeared to have deeply stained nuclei and congested sinusoids. In addition, centrilobular necrosis, cellular hypertrophy and vacuolization were observed (Figures 3C, D).

Supplementation with PA alleviated these pathological changes in the form of clear central veins and sinusoids. The hepatocytes appeared normal on the cytoplasmic and nuclear levels (Figures 3E, F). Also, the co-administration of PA and APAP led to improvements in the pathological changes in the form of minimal congestion of the central vein. Most of the hepatocytes restored the normal appearance of the cytoplasm and nuclei. However, the perivascular cells still showed vacuolated cytoplasm with darkly stained nuclei (Figures 3G, H).

### Histopathological evaluation of hepatic inflammation, necrosis, vacuolation and vascular congestion in the study groups

Table 4 provided a descriptive analysis of various histopathological changes exhibiting the negative effects of APAP and the positive effects of PA. The scores of the histopathological changes (= sum of scores in the 5 examined fields) were significantly higher in APAP group (*p* < 0.001) as compared to control group. In contrary, PA group showed a significant decrease in the histopathological score correlated to APAP group (*p* < 0.001). Coadministration of PA caused a significant drop in the histopathological score as compared with APAP group (*p* = 0.008).

**TABLE 4:** Results of Histopathological scoring in all the study groups.

	Control	APAP	PA	APAP + PA	*p*-value
Mean ± SD	0.67 ± 0.81	3.5 ± 0.54^a, c^	0.67 ± 0.51^b^	1.83 ± 0.75^c^	0.001*

Comparison between groups was performed via one-way ANOVA, followed by a *post hoc* Games Howell test. a: significance between the control and APAP, groups (*p*-value = 0.001), b: significance between the PA, and APAP, groups (*p*-value = 0.001), c: significance between the APAP and APAP + PA groups (*p*-value = 0.008). * Indicated significance among the 4 studied groups.

### Perl’s prussian blue stain among different experimental groups for the detection of iron in the hepatic tissue

**FIGURE 4:**
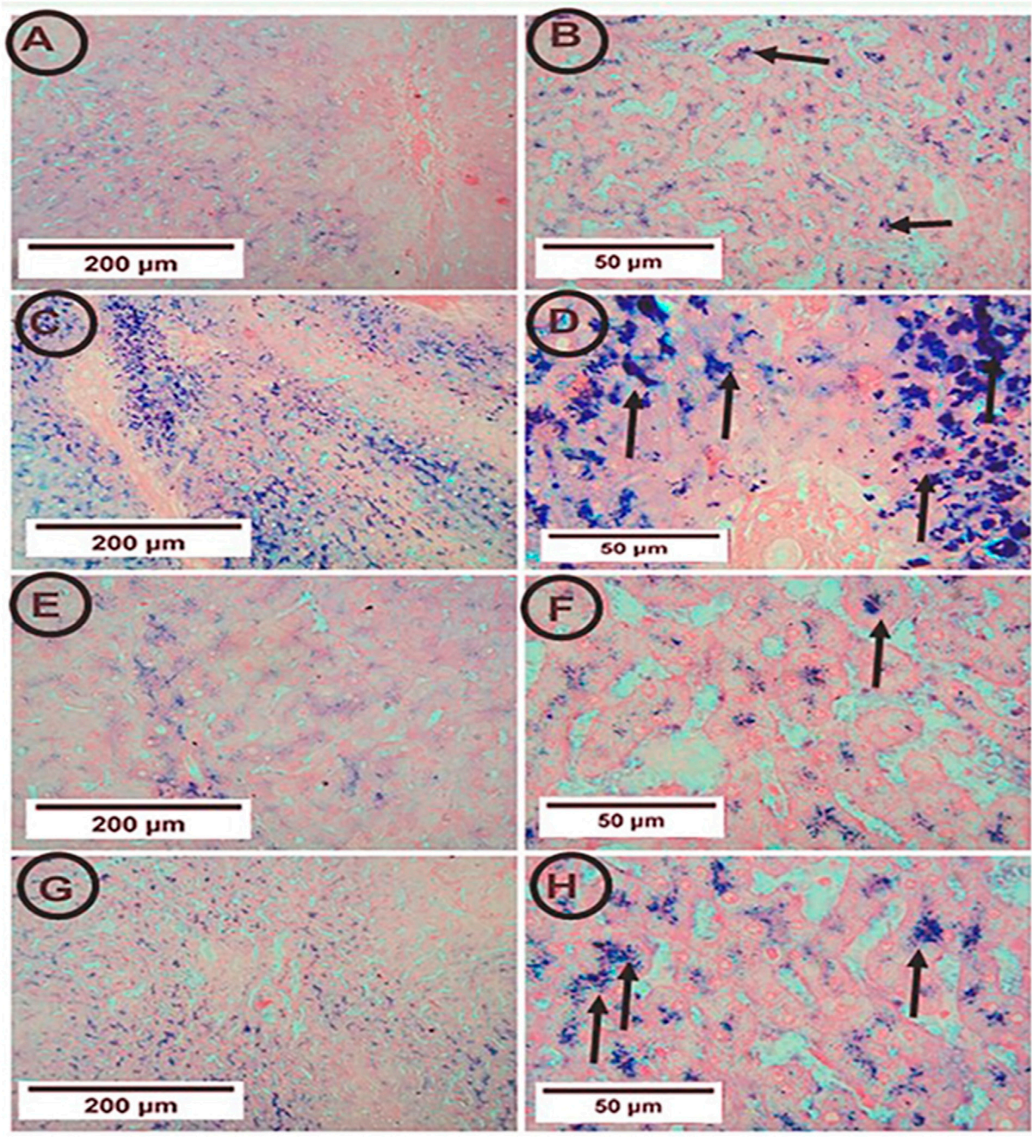
Photomicrographs of hepatic tissue in the study groups (6 mice in each group). **(A,B)** the control group showed few blue granules of hemosiderin in hepatocytes (arrows). **(C,D)** the APAP group showed many blue granules of hemosiderin in hepatocytes (arrows). **(E,F) **the PA group showed few blue granules of hemosiderin in hepatocytes (arrows). **(G,H)** the APAP + PA group showed fewer blue granules of hemosiderin in hepatocytes more or less like the control group (arrows) (Perl’s Prussian blue iron stain: A, C, E, G X 100 – B, D, F, H X 400). Abbreviations: APAP, acetaminophen; PA, phytic acid.

The control rats showed few blue granules of hemosiderin in hepatocytes. On the contrary, the APAP group showed enhancement of blue granules of hemosiderin in hepatocytes, indicating iron deposition. The co-administration of PA and APAP resulted in fewer blue granules (Figure 4).

### Immunohistochemical expression of *SIRT-1* in the hepatic tissue among different experimental groups

Both the control and PA groups showed positive *SIRT-1* expression, but the APAP group showed weak positive *SIRT-1* expression. The co-administration of PA with APAP led to strong positive *SIRT-1* expression (Figure 5).

**FIGUR 5:**
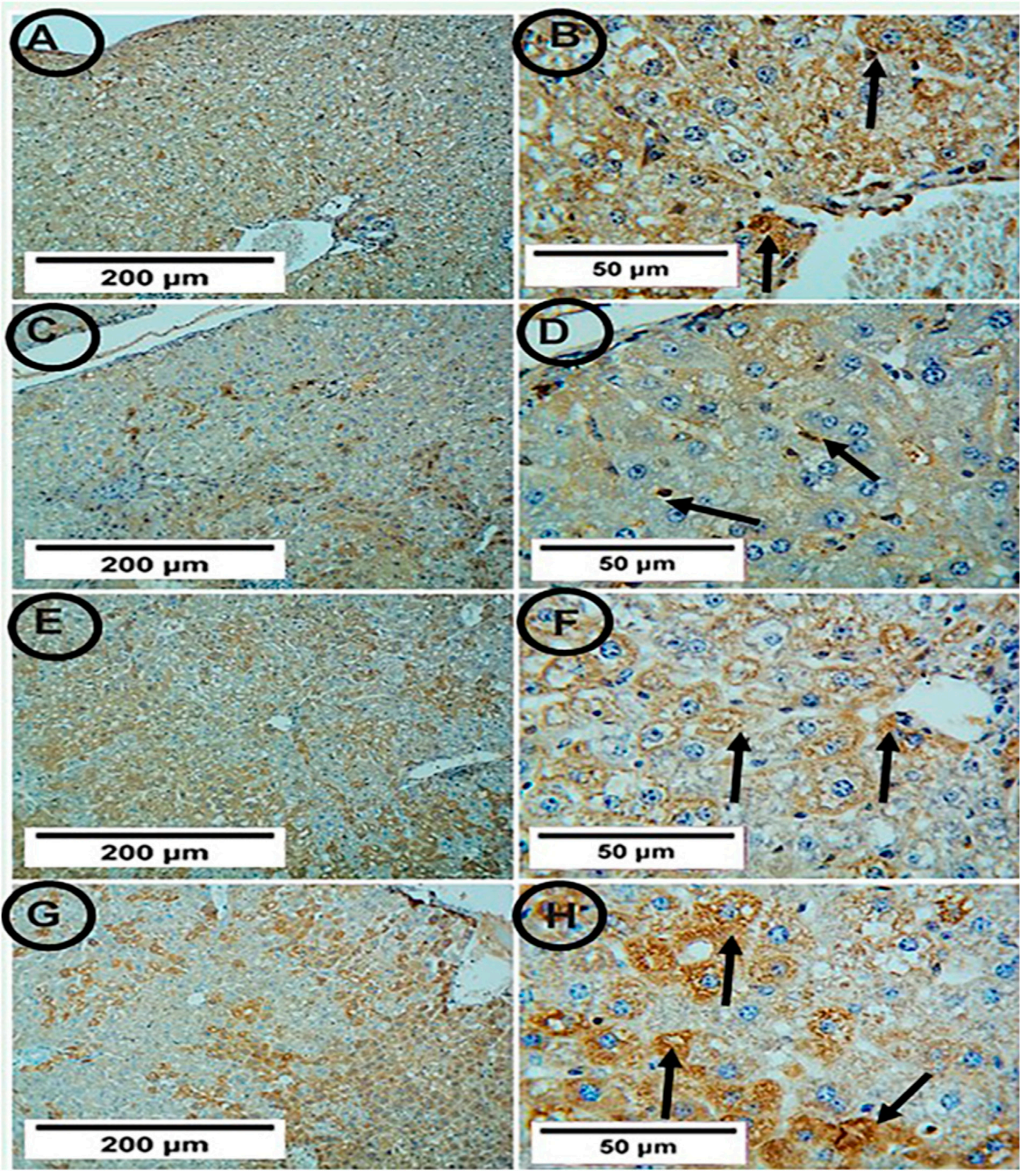
Photomicrographs of hepatic tissue in the study groups (6 mice in each group). **(A,B)** the control group showed positive *SIRT-1* expression (arrows). **(C,D) **the APAP group showed weak positive *SIRT-1* expression (arrows). **(E,F) **the PA-treated group showed positive *SIRT-1* expression (arrows). **(G,H)** the APAP + PA group showed strong positive *SIRT-1* expression (arrows) (*SIRT-1*, A, C, E, G X 100 – B, D, F, H X 400). Abbreviations: APAP, acetaminophen; PA, phytic acid; *SIRT-1*, sirtuin-1.

### Results of measurement of area percentage positive *SIRT*-1 reaction in hepatic tissue

**FIGURE 6:**
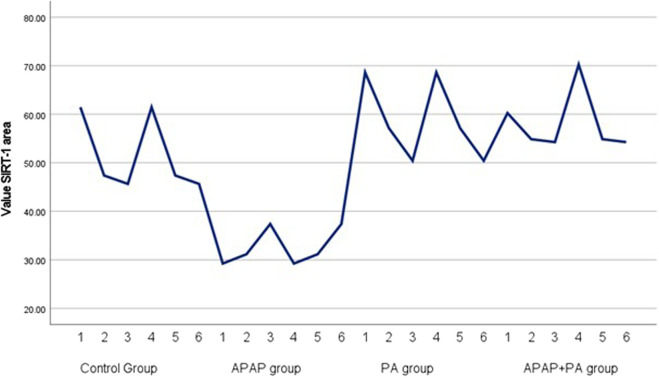
Simple line graph for individual values of *SIRT*-1 area percentage in each group (6 mice in each group). Comparisons between groups were performed via one-way ANOVA followed by a post hoc Games Howell test. Abbreviations: APAP; acetaminophen, PA, phytic acid, SIRT-1, sirtuin-1.

The APAP group showed a significant decrease in the percentage of *SIRT1* positive reaction when compared to the control group (*p*-value ≤ 0.001). The co-administration of PA and APAP resulted in a significant increase in *SIRT-1* area percentage when compared to the APAP group (*p*-value ≤ 0.001) (Figure 6).

**FIGURE 7:**
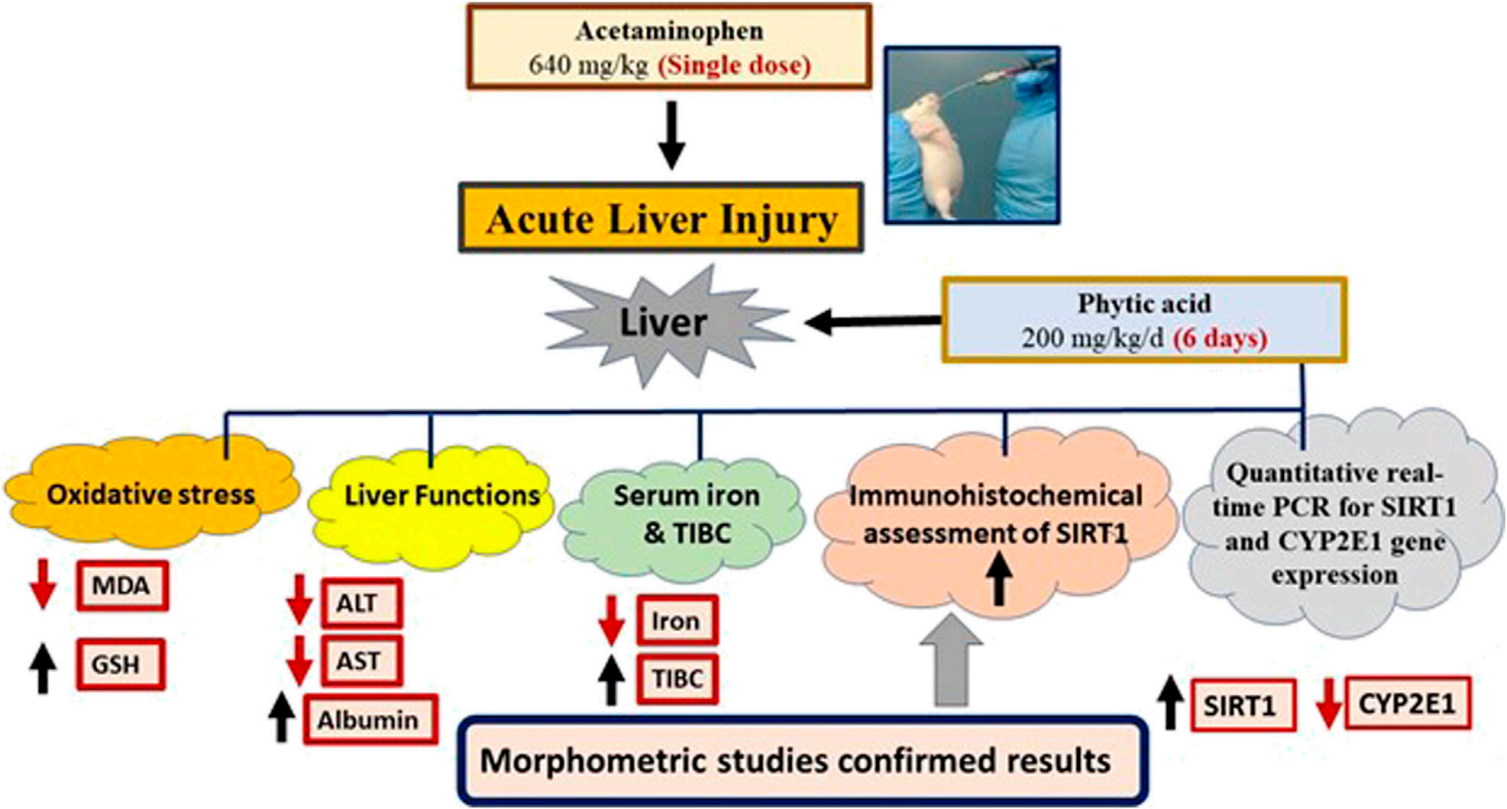
Graphical abstract. Phytic acid protected the mice liver for acetaminophen-induced injury via regulation of oxidative stress, liver functions, SIRT-1 and CYP2E1 gene expression. MDA, Malondialdehyde; GSH, Reduced glutathione; ALT, Alanine transaminase; AST, Aspartate transaminase; TIBC, Total iron binding capacity. SIRT1, Sirtuin-1; CYP2E1, Cytochrome P450 family 2 subfamily E member 1

All results were summarized in a graphical abstract (Figure 7).

## Discussion

Acetaminophen is one of the most widely used analgesic and antipyretic over-the-counter drugs in the world (Lee, 2008). Although APAP is considered safe at therapeutic doses, its overdose can induce severe liver toxicity and even death (Liao et al., 2023). APAP overdose hepatotoxicity is the leading cause of acute liver failure (Yoon et al., 2016). APAP toxicity could be due to the ingestion of a single overdose or too frequent doses with therapeutic intent (Naqvi et al., 2010). N-acetyl cysteine is the mainstay of treatment for APAP-induced hepatotoxicity; however, this medication has some drawbacks in terms of side effects and a narrow therapeutic window (Bateman et al., 2014). Therefore, the establishment of novel therapeutic approaches is required.

It has been documented that PA administration provided protection through metal chelation and its antioxidant properties (Kumar et al., 2010). Thus, the present study demonstrated the efficacy of PA in combating the APAP-induced hepatotoxicity through its iron chelating activity and antioxidant effect. It was ensured that the toxic dose of acetaminophen was reached by estimating the serum levels of APAP using HPLC. In the current study, the estimated measured levels of APAP were indicative of reaching toxicity levels, as previously reported by Dai et al. (2006); Dargue et al. (2020).

The present data based on APAP-induced hepatotoxicity in mice identified that PA treatment for 7 days at a dose of 200 mg/kg/day has critical actions in modulating APAP-induced liver toxicity in mice. The evidence for this is provided by evaluating the liver function biomarkers, *CYP2E1* gene expression, iron overload, oxidative stress, *SIRT-1* expression and hepatic architecture damage associated with APAP overdose.

Results of the current study revealed that there was a deteriorating effect of APAP on liver functions, as determined by significantly elevated liver enzymes and decreased albumin level in mice serum in the APAP group when compared to the control group. PA treatment produced a statistically significant decrease in liver enzymes and an increase in the albumin level compared to the APAP group.

The effect of PA on the modulation of liver function was in harmony with the results of Al-Fatlawi and Al-Shammari (2017), which supported the protective effects of PA against methotrexate-induced acute liver injury. Xu et al. (2023) also demonstrated the PA protective effects and modulation of liver functions in CCL4-induced hepatotoxicity in mice.

It was well established that the hepatotoxicity of APAP begins with the metabolic conversion of APAP to its reactive metabolite (N/-acetyl-p-benzoquinone imine, or NAPQI) (Wallace, 2004). *CYP2E1* catalyzes the oxidation of APAP to NAPQI, a highly reactive metabolite inducing severe massive hepatocellular necrosis (Ramachandran and Jaeschke, 2020). *CYP2E1* knockout mice showed resistance to the APAP high dose-associated hepatotoxicity, suggesting that *CYP2E1* is the major cytochrome P450 enzyme participating in APAP metabolism and toxicity (Cheung et al., 2005). In addition to the enhancement of NAPQI production by *CYP2E1* in APAP toxicity, CYP enzymes, notably *CYP2E1*, generate ROS and lipid peroxidation (Veith and Moorthy, 2018).

As *CYP2E1* is the major source of NAPQI, an evaluation of the *CYP2E1* expression level was done in this study. APAP treatment caused a significant increase in *CYP2E1* mRNA level compared to the control group and this finding was in accordance with the reported results that *CYP2E1* could play a major role in APAP-induced cytotoxicity (Knockaert et al., 2011; Chen et al., 2020). Administration of PA in APAP-induced hepatotoxicity in this study produced a significant reduction in the *CYP2E1* mRNA level. The ability of the decreased *CYP2E1* to suppress APAP-induced hepatotoxicity was previously reported by Lee et al. (1996), who demonstrated resistance to APAP-induced hepatotoxicity in *CYP2E1* knockout mice, indicating that CYP2E1 is the key enzyme responsible for the metabolic conversion of APAP into the active hepatotoxic NAPQI.

As previously mentioned, iron plays a role in APAP-caused hepatotoxicity. Iron is considerably more toxic to cells expressing *CYP2E1* than to cells not expressing this enzyme, and it is suspected that low concentrations of iron may act as a protective factor for *CYP2E1*-induced injury. The possibility of minimizing iron intake or removing iron may prove beneficial for the treatment of liver injury and it is clearly an area that deserves further research (Cederbaum, 2003).

For investigating the pathogenesis and treatment of APAP-induced hepatotoxicity, the role of iron was evaluated as was whether PA played a role in the treatment of APAP-induced hepatotoxicity in light of its importance as an iron chelator and its ability to block the generation of iron-driven hydroxyl radicals as well as the suppression of lipid peroxidation (Omoruyi et al., 2013). Because the SI and TIBC are the principal tests used in the evaluation of iron burden, those tests were evaluated in different groups in the present study. APAP treatment caused a significant increase in SI and a significant decrease in the TIBC in comparison with the control group. PA treatment produced the reverse effect. This confirms the role of iron overload in the pathogenesis of APAP-induced hepatotoxicity and the role of PA in alleviating the iron burden. This finding was similar to that of a previous study by Mahmoud et al. (2018), which indicated that high levels of dietary phytate reduced serum iron levels.

The serum iron could reflect hepatic iron accumulation, which is considered an important factor in the progression of liver fibrosis (Metwally et al., 2004). For confirmation of the iron accumulation in the hepatic tissue induced by APAP toxicity, Perl’s Prussian blue stain among different experimental groups was done. The APAP group showed an enhancement of blue granules of hemosiderin in the hepatocytes, indicating iron deposition. On the contrary, PA supplementation led to a reduction in the blue granules, indicating less iron deposition.

On exposure to a toxic dose of APAP, ferrous iron is released into the cytosol from the ruptured lysosomes (Kon et al., 2010). Cytosolic iron overload can result in the generation of ROS mediated by the Fenton reaction, a process that could aggravate the oxidative stress already induced by APAP (Jomova and Valko, 2011). Thus, in this study, we evaluated lipid peroxidation by measuring the level of MDA and evaluated antioxidant cellular activity by measuring the GSH level in mice liver tissue homogenate.

The current results showed that APAP treatment induced a statistically significant increase in MDA and a decrease in GSH levels compared to the control group, while PA produced a significant improvement in oxidative stress by decreasing the MDA level and increasing the GSH level compared to the APAP group. In agreement with this, the ability of PA to protect cells against oxidative stress has been associated with inhibited ROS generation, increased GSH levels, and decreased lipid peroxidation (MDA), as shown in several studies (Lee et al., 2005; Abdel-Hamid et al., 2007; Foster et al., 2017; Da Silva et al., 2019). Bhowmik et al. (2017) demonstrated that PA could reduce the elevated level of oxidative stress caused by an iron-overloaded liver in thalassemia through the amelioration of inflammatory responses through the suppression of ferritin levels.

An increased ROS level can directly or indirectly control the *SIRT-1* enzyme (Salminen et al., 2013). *SIRT-1* has a crucial role in modulating multiple physiological processes. It could be responsible for maintaining homeostasis between DNA damage, senescence, and apoptosis under oxidative stress (Singh et al., 2018; Jiao and Gong, 2020). In response to oxidative stress, *SIRT1* is redistributed at the chromatin level, causing deregulation at the transcription level and DNA damage (Oberdoerffer et al., 2008). Moreover, there is a mutual interplay between iron and sirtuins, in which iron overload results in a significant decrease in *SIRT-1* mRNA expression and induces apoptosis (Tinkov et al., 2021). On the other hand, *SIRT-1* can regulate cellular survival through its role in controlling iron hemostasis. *SIRT-1* can decrease iron uptake from the extracellular environment by stabilizing iron regulatory protein, which is responsible for iron transport into the cell. In addition, *SIRT-1* can increase ferritin expression, thus reducing serum iron (Farghali et al., 2019; Su et al., 2021).

In the current study, the *SIRT-1* expression level in mice liver was evaluated. The APAP group showed a significant decrease in *SIRT-1* expression level compared to the control group. This finding was in accordance with previously reported results (Wang et al., 2020; Hu et al., 2021). On the other hand, PA administration significantly increased *SIRT-1* expression levels in APAP-induced hepatotoxicity. This effect can be explained by three mechanisms: First, the ability of PA to chelate iron, as Tinkov et al. (Tinkov et al., 2021) reported that iron overload decreased *SIRT*-1 gene expression. Second is the role of PA in attenuating oxidative stress, which in turn may lead to increased *SIRT-1* expression (Anekonda et al., 2011). Finally, the direct effect on PA in *SIRT-1* upregulation (Zhang and Tang, 2023).

The alleviating effect of PA was confirmed by hepatic structural examination, which revealed structural improvement in the PA group compared to the APAP group. This result was in agreement with (Xu et al., 2023), who previously demonstrated the ability of PA to alleviate structural liver damage in CCL4-induced hepatoxicity in mice.

## Conclusion

The present study pointed to the protective effect of PA against APAP-induced hepatotoxicity in mice through ameliorating the functional, biochemical and morphological changes of APAP-induced hepatotoxicity. PA’s beneficial effects might be attributed to iron chelation, decreased *CYP2E1* mRNA expression, increased *SIRT*-1 expression at both mRNA and protein levels, and decreasing oxidative stress.

Further researches are needed to confirm PA safety and efficacy on other animal species in either APAP induced hepatotoxicity or other hepatotoxicity studies and further extension on clinical phases based on previous experimental studies.

## Limitations of the study

The findings of this study could be considered with a few limitations. Estimation of NAPQI, different sulfate and glucuronide metabolites in hepatic tissue and serum, and a survival study of APAP have not been evaluated in this study which could add more confirmation for APAP toxicity and PA role in APAP-induced hepatotoxicity. Also, only 1 dose of PA was tested and dose-related effects could strengthen the case for the potential therapeutic effect of PA.

## Data Availability

The original contributions presented in the study are included in the article/Supplementary materials, further inquiries can be directed to the corresponding author.

## Abbreviations

APAP, acetaminophen; *CYP2E1*, Cytochrome P450 family 2 subfamily E member 1; NAPQI, N-acetyl-p-benzoquinone imine; GSH, Reduced glutathione; *SIRT-1*, Sirtuin-1; PA, Phytic acid; ALT, Alanine transaminase; AST, Aspartate transaminase; SI, Serum iron; TIBC, Total iron-binding capacity (TIBC); MDA, Malondialdehyde; GAPDH, Glyceraldehyde-3-phosphate dehydrogenase; HPLC, High-performance liquid chromatography; qRT-PCR, Quantitative real-time polymerase chain reaction; CT, Cycle threshold.
